# Genetic manipulation of *Soc1*‐like genes promotes photosynthesis in flowers and leaves and enhances plant tolerance to high temperature

**DOI:** 10.1111/pbi.13432

**Published:** 2020-06-28

**Authors:** Guogui Ning, Xu Yan, Hai Chen, Ruijie Dong, Weiqing Zhang, Ying Ruan, Wenen Wang, ManZhu Bao, Henry Daniell, Shuangxia Jin

**Affiliations:** ^1^ Key laboratory of Horticultural Plant Biology Ministry of Education Huazhong Agricultural University Wuhan China; ^2^ State Key Laboratory of Agricultural Microbiology Huazhong Agricultural University Wuhan China; ^3^ Department of Biochemistry School of Dental Medicine University of Pennsylvania Philadelphia USA; ^4^ National key laboratory of Crop Genetics and Improvement College of Plant Science & Technology Huazhong Agricultural University Wuhan China

**Keywords:** *Soc1‐like* genes, photosynthesis, flowers and leaves, heat tolerance, tobacco, petunia

The rapid rise in mean global temperature as a result of global warming threatens plant productivity (Li *et al*., 2015). Chloroplasts and chloroplast proteins are associated with environmental stresses (Alexia *et al.,* 2019; Hong *et al*., 2020). Many heat‐shock proteins (HSPs) associate with chloroplast development and improve plant tolerance to heat stress at a high temperature (Shen *et al*., 2015; Zhong *et al*., 2013), whereas no gene is reported to promote chloroplast development and enhance tolerance to high temperature synchronously. The impact of high temperature on chloroplast is of particular significance since photosynthesis is often inhibited before other cell functions are impaired (Zhang *et al*., 2010). Thus, promoting chloroplast biogenesis and photosynthesis is a potential method to enhance heat tolerance of plants. We previously found that overexpression of *SOC1* or *SOC1‐like* genes in heat‐stressed plants induces chloroplast biogenesis in petals (Wang *et al*., 2019). However, it is unknown whether the photosynthesis apparatus is impaired and whether the plant thermotolerance is enhanced in transgenic plants. In our present study, the transplastomic (harbouring *GFP* reporter gene driven by *psbA* promoter of chloroplast), multigene transgenic tobacco (*Fbp21* gene was introduced to the genome of the pure line of *GFP* transplastomic tobacco‐labelling nFbp21*pGFP) and transgenic petunia‐overexpressing *FBP21* gene were produced by chloroplast and nuclear transformation. Additionally, the transgenic plants (Fbp21‐labelling F21 and Fbp21*22‐labelling F21_22 in this paper) harbouring *SOC1‐like* genes and RNA‐Seq data of petals, previously reported (Wang *et al*., 2019), were also integrated. Finally, a series of experiments related to RNA sequencing in leaves, biological and physiological, anatomical and phenotypic determination were undertaken.

When plants were grown at high temperature (40°C days/28°C nights), it showed that only nonphotosynthetic plastids containing plastoglobules were seen in pink petals of control tobacco plants. We observed morphologically normal chloroplasts in green petals of the *SOC1‐like* gene transgenic tobacco plants (Figure 1a). Chloroplasts in green petals of nFbp21*pGFP transplastomic tobacco were observed to emit red and green fluorescence simultaneously at high temperature (Figure 1b). It indicates that chloroplast genes were expressing in these heat stress‐induced plastids in petals. Maximum photochemical efficiency values (*F_v_*/*F_m_*) determination also showed that photosynthesis took place in chloroplast‐containing petals (Figure 1c and d). Most of the photosynthesis genes were dramatically up‐regulated in chloroplast‐containing green petals (Figure 1e). Immunoblot analysis also showed many photosynthesis associated proteins were synthesized in green petals (Figure 1f).

**Figure 1:**
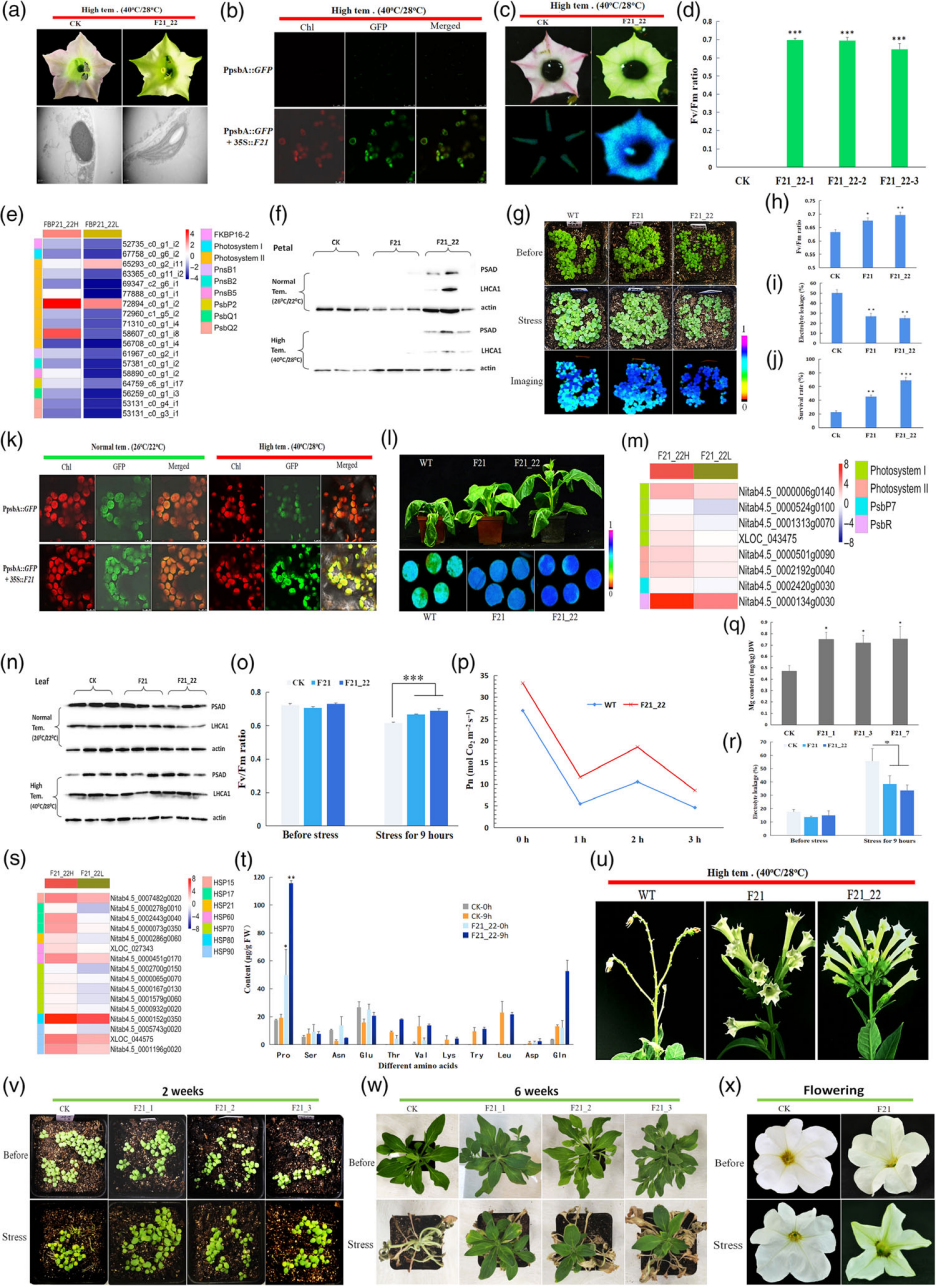
*Soc1‐like* genes promote photosynthesis and improve heat tolerance in plant. (a) Colour and anatomy of transgenic tobacco grown at higher temperature. (b) Red and green fluorescence in heat stress petals. (c‐d) *F_v_/F_m_* values in control and transgenic tobacco petals. (e) Heat map shows expression profiles of differently expressed genes (DEGs) associated with photosynthesis in green petals. (f) Immunoblot analyses of photosynthesis proteins (PSAD and LHCA1) in transgenic tobacco petals. (g) Phenotypes of 2‐week‐old transgenic tobacco suffering from 4 days of heat stress (45°C day/28°C night). (h‐j) *F_v_/F_m_* values (h), EL levels (i) and seedling survival rate(j)—scored to those recovered at 3 days (26°C day/22°C night)—of control and transgenic tobacco after heat stress. (k) Chloroplasts in leaves of the *GFP* and nFBP21*p*GFP* transgenic tobacco grown at normal and high temperature. (l) Phenotypes of 6‐week‐old transgenic tobacco after heat stress (9 h 45°C). (m) Heat map shows expression profiles of DEGs associated with photosynthesis in the leaves of transgenic tobacco plants grown in normal and high temperature. (n) Immunoblot analyses of photosynthesis proteins in tobacco leaves. (o) Leaf *F_v_/F_m_* values of 6‐week‐old control and transgenic tobacco before and after stress. (p) Leaf Pn values of wild type and transgenic tobacco at different times of heat stress. (q‐r) Magnesium content (r) and EL levels (r) in leaves of 6‐week‐old control and transgenic tobacco. (s) Heat map shows expression profiles of DEGs encoding heat‐shock proteins in leaves of transgenic tobacco grown at normal and high temperature. (t) Amino acid content of leaves in transgenic tobacco. (u) Phenotype of transgenic tobacco grown at high temperature. (v) Phenotypes of 2‐week‐old *Fbp21* transgenic petunia acclimated for 5 days at high temperature (45°C/30°C). (w) Phenotypes of 6‐week‐old transgenic petunia before and after heat stress. (x) Light green petals in heat‐treated *Fbp21* transgenic petunia. Note: Error bars represent ± SE (*n* = 3). Asterisks indicate significant differences (** *P* < 0.01, *** *P* < 0.001).

Heat‐resistant assay showed that the *SOC1‐like* gene transgenic tobacco (F21and F21_22) was substantially different from the control tobacco in their tolerance to prolonged extreme heat stress. For 2‐week‐old tobacco plants, more light yellow seedlings were seen in the wild‐type tobacco than in transgenic lines after heat stress (Figure 1g). *F_v_/F_m_* values in transgenic lines were also notably higher than that of wild type (Figure 1g and h). The lower electrolyte leakage (EL) (Figure 1i) and higher survival rate (Figure 1j) suggest that these 2‐week‐old transgenic tobacco had enhanced heat tolerance. When plants were grown at normal temperature, more chloroplasts in cells of leaves of the transgenic tobacco (including F21 or F21_22 tobacco) also observed in the nFbp21*pGFP transplastomic tobacco according to red and green fluorescence compared to *GFP* transplastomic tobacco (Figure 1k). These results suggest that overexpression of *Soc1‐like* genes promotes chloroplast biogenesis in transgenic leaves. When grown at high temperature, the leaf chloroplasts of the transgenic tobacco (also seen in nFbp21*pGFP) maintained normal appearance and orderly distribution and emitted more green fluorescence (Figure 1k). These observations indicate that the chloroplast genes can normally express at high temperature, whereas the chloroplasts in leaves of control tobacco became swollen, globular and irregular. It was consistent with what reported by Kwon and colleagues in *GFP* transplastomic tobacco (Kwon *et al*., 2013). The structural changes of chloroplasts and their scattered distribution in control tobacco (Figure 1k) suggest higher instability of varied cell membranes and cell damages by heat at high temperature.

The response to high temperature of 6‐week‐old tobacco plants was also markedly different between control and transgenic tobacco (Figure 1l). Many photosynthesis genes were dramatically up‐regulated in leaves of transgenic plants growing at high temperature (Figure 1m). Immunoblot analysis showed that photosynthesis‐associating proteins were accumulated in transgenic plants (Figure 1n). Under continuous heat stress (45°C for 9 h), *F_v_*/*F_m_* values were also significantly higher in leaves of transgenic tobacco plants (Figure 1o). A time series of net photosynthetic rate (Pn) determination indicated that the leaf Pn rate of transgenic tobacco plants was higher than that of wild type (Figure 1p). Magnesium is part of the chlorophyll and essential for photosynthesis (Leonard, 1954), and higher magnesium content was also detected in leaves of F21 transgenic tobacco plants (Figure 1q). Taking together, these results suggested that *SOC1‐like* gene transgenic tobacco plants possess enhanced photosynthetic capacity under heat stress conditions.

Leaf EL value of 6‐week‐old transgenic tobacco was lower than that of wild type after heat stress (Figure 1r). RNA‐seq analysis showed that genes encoding heat‐shock proteins were also greatly up‐regulated in leaves of transgenic tobacco plants under heat stress (Figure 1s). GC‐MS analysis showed the proline content was significantly higher in *SOC1‐like* gene transgenic tobacco plants (Figure 1t). It is worth noticing that during budding phase, the transgenic tobacco plants were not impaired and flowered normally under heat stress (12‐h light cycle for 15 days). In contrast, wild‐type tobacco plants did not flower or flowered poorly under the same heat stress condition (Figure 1u).

Additionally, the transgenic petunia‐overexpressing *Fbp21* gene was also more heat tolerant at varied growth phases than wild‐type petunia plants (Figure 1v and w). The highest survival rate (96.60%) was recorded for 2‐week‐old transgenic petunia after heat stress (Figure 1v). For 6‐week‐old petunia seedlings, none of the wild‐type petunia seedlings survived after 3 days under high temperature (Figure 1w). Similar to tobacco‐overexpressing *SOC1‐like* genes, the transgenic petunia‐overexpressing *Fbp21* gene also produced light green petals under heat stress (Figure 1x).

In an earlier study, we reported for the first time that *SOC1‐like* genes promote chloroplast biogenesis in heat‐stressed petals (Wang *et al*., 2019). In the present study, we show that cell containing increased number of chloroplasts is observed more frequently in leaves of *Soc1‐like* gene transgenic plants than in wild‐type plants and that *SOC1‐like* genes up‐regulate photosynthesis and heat‐shock‐associated genes, improve plant photosynthesis and alleviate heat stress damage to the chloroplast. Our results demonstrated that the super plants having chloroplast‐containing petals, higher chlorophyll contents, increasing photosynthesis and enhancing heat tolerance could be synchronously achieved by genetic engineering. We showed that plant flowers can perform photosynthesis to further improve carbon utilization efficiency under heat stress and that overexpression of *SOC1‐like* genes reduce the deleterious effects of heat stress on chloroplast and enhance photosynthesis in plants. Our observation provides a novel insight into the crosstalk mechanism between high temperature, plant functional chloroplast biogenesis, plant photosynthesis and plant heat tolerance. Producing heat‐tolerant plants will be of great ecological and economic significance under the increasing threat of global warming.

## Competing financial interests

The authors declare no competing financial interests.

## Author contributions

G.N. designed the experiments and wrote the manuscript. G.N., M.B., H.D and S.J. supervised the research and finally reviewed the paper. H.C., R.D., W.Z., Y.R and W.W conducted the experiments. X.Y analysed the RNA‐Seq data. All authors participated in data interpretation.
